# Application of 3D printing to prototype and develop novel plant tissue culture systems

**DOI:** 10.1186/s13007-017-0156-8

**Published:** 2017-01-19

**Authors:** Mukund R. Shukla, Amritpal S. Singh, Kevin Piunno, Praveen K. Saxena, A. Maxwell P. Jones

**Affiliations:** Department of Plant Agriculture, Gosling Research Institute for Plant Preservation, University of Guelph, 50 Stone Rd. E, E.C. Bovey Building Room 4221, Guelph, ON N1G 2W1 Canada

**Keywords:** 3D printing, Prototyping, Plant tissue culture, Micropropagation, Light quality, LED lighting system, Culture vessel design

## Abstract

**Background:**

Due to the complex process of designing and manufacturing new plant tissue culture vessels through conventional means there have been limited efforts to innovate improved designs. Further, development and availability of low cost, energy efficient LEDs of various spectra has made it a promising light source for plant growth in controlled environments. However, direct replacement of conventional lighting sources with LEDs does not address problems with uniformity, spectral control, or the challenges in conducting statistically valid experiments to assess the effects of light. Prototyping using 3D printing and LED based light sources could help overcome these limitations and lead to improved culture systems.

**Results:**

A modular culture vessel design in which the fluence rate and spectrum of light are independently controlled was designed, prototyped using 3D printing, and evaluated for plant growth. This design is compatible with semi-solid and liquid based culture systems. Observations on morphology, chlorophyll content, and chlorophyll fluorescence based stress parameters from in vitro plants cultured under different light spectra with similar overall fluence rate indicated different responses in *Nicotiana tabacum* and *Artemisia annua* plantlets. This experiment validates the utility of 3D printing to design and test functional vessels and demonstrated that optimal light spectra for in vitro plant growth is species-specific.

**Conclusions:**

3D printing was successfully used to prototype novel culture vessels with independently controlled variable fluence rate/spectra LED lighting. This system addresses several limitations associated with current lighting systems, providing more uniform lighting and allowing proper replication/randomization for experimental plant biology while increasing energy efficiency. A complete procedure including the design and prototyping of a culture vessel using 3D printing, commercial scale injection molding of the prototype, and conducting a properly replicated experiment are discussed. This open source design has the scope for further improvement and adaptation and demonstrates the power of 3D printing to improve the design of culture systems.

**Electronic supplementary material:**

The online version of this article (doi:10.1186/s13007-017-0156-8) contains supplementary material, which is available to authorized users.

## Background

Plant tissue culture is the aseptic culture of cells, tissues, organs or whole plants under controlled nutritional and environmental conditions, allowing the growth and development of the cells or tissues to be manipulated for a variety of applications. These techniques provide powerful tools to study fundamental processes in plants and form the basis of many biotechnological applications. One of the most important commercial applications of plant tissue culture is large-scale plant multiplication for the production of insect/disease/virus free plants, particularly valuable for vegetatively propagated plants such as potato, garlic, banana, sugar cane, orchids and fruit trees. The value of such an approach is exemplified in the seed potato industry, where the use of certified disease free propagules has eradicated a number of diseases from various regions and helped limit the spread of others [1, 2]. Using this approach, a single explant can be multiplied to produce several thousand plants in a relatively short time period and little space on a year round basis. Despite the importance of plant tissue culture and micropropagation in several sectors, the general techniques used for in vitro propagation have not changed much in recent years, with little development or innovation in vessel design or culture systems.

Among the environmental conditions affecting plant growth and development, light is known to have profound effects [3]. Light provides energy through photosynthesis and acts as a signalling mechanism through a variety of light receptors. The fluence rate, spectrum, and duration of light/dark form the key quality attributes that affect photosynthesis and photomorphogenesis. Modulation of light quality is therefore employed widely to enhance plant growth, propagation, and production systems [4, 5]. Though light quality is of key significance, experimentation with light qualities affecting in vitro growth of plants presents a number of challenges related to control over the light spectrum produced and difficulties in proper replication and experimental design.

**Fig. 1:**
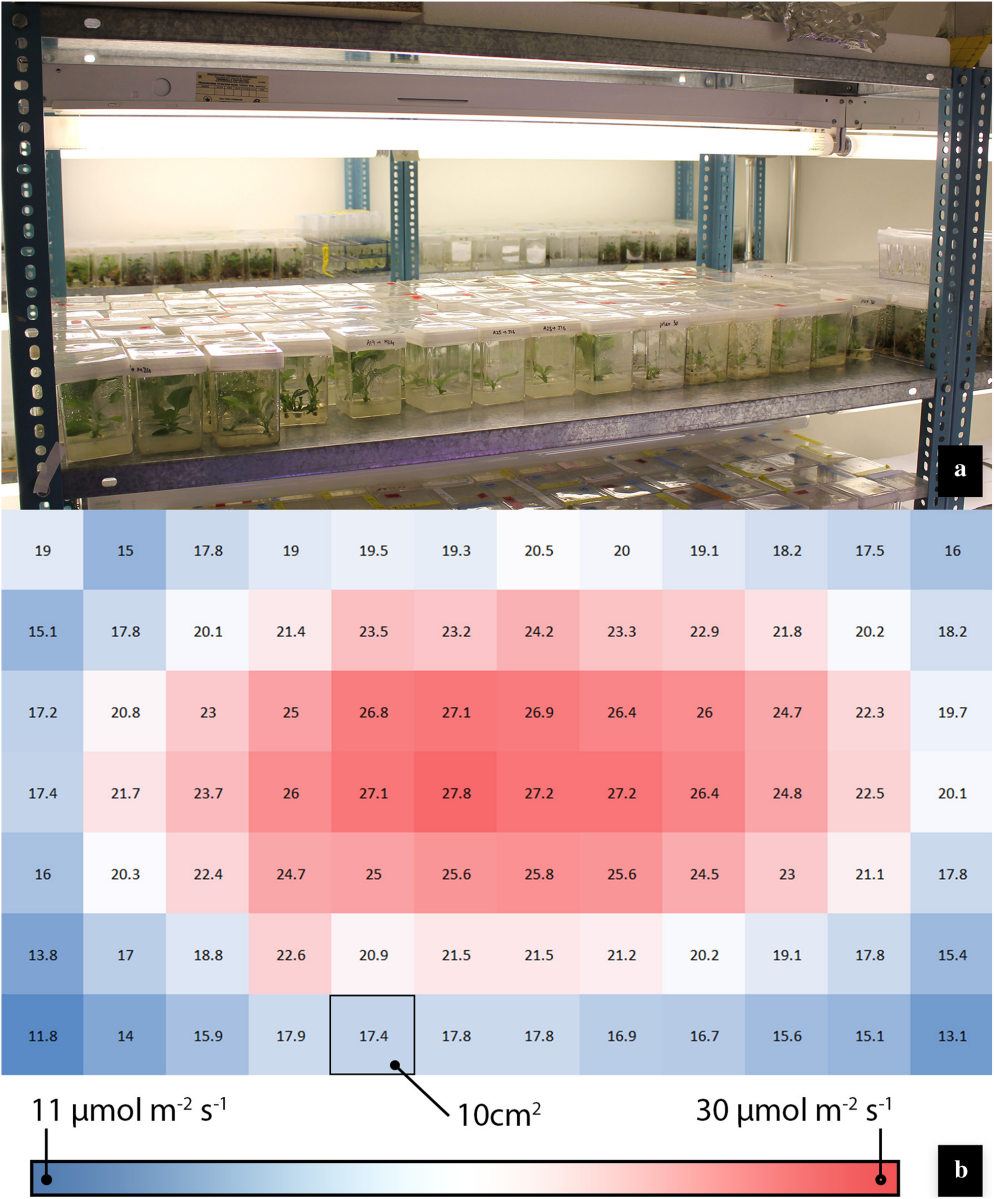
A typical tissue culture room shelf (120 × 60 × 40 cm) with two florescent bulbs on a ballast at the center of the shelf (**a**), Heat map of light fluence rate (**b**). *Each square* represents a 10 cm^2^ area measured from the center with a light meter 31 cm from the light

Fluorescent lamps are currently the most common light source used and consume approximately 65% of total electricity in tissue culture labs [6]. In most plant tissue culture facilities fluorescent lights are fixed on the shelves of culture racks at a particular height, and light distribution on any given shelf is not completely uniform, as demonstrated in Fig. 1. Further, most fluorescent lights have sub-optimal spectra for plant growth and the spectra and fluence rate change as the bulbs age due to cathode decay and a reduction in energy transferred through the mercury vapour. The spectra of light can also vary across the shelf, resulting in different proportions of red and blue wavelengths [variation in correlated colour temperature (CCT) values over a shelf; Additional file 1: Figure S1]. From the perspective of experimental biology, one of the greatest drawbacks of using fluorescent lighting is that each bulb generally provides light for an entire shelf such that proper replication and randomization for proper experimental design takes many shelves/chambers and is often not practical. As such, much of the research on the effects of light fluence rate/quality have been conducted using pseudo-replicates that do not meet the strict assumptions required for proper statistical analysis.

The development of high fluence rate LEDs provides a promising alternate light source for plant growth in controlled environments [6]. In contrast to fluorescent lights, LEDs are highly modular and can be more evenly distributed to give more uniform lighting, they often have a very narrow emission wavelength that is stable over time, can be combined to produce a desired spectrum, are more energy efficient, and are longer lasting. In addition to increasing the energy efficiency, the ability to select and control the spectrum could greatly improve plant tissue culture systems as both fluence rate and quality of light can influence plant growth and development. The photosynthetic ability of in vitro plantlets [7] can be improved by changing the light fluence rate and quality in the growth environment [8]. Light-emitting diodes (LED) have been used to accelerate plantlet growth and their effects on chlorophyll synthesis [9, 10], photosynthesis [11, 12], and morphogenesis [5, 9, 13–15] have been studied in a variety of species.

While LEDs have been used to improve plant growth, they are generally used as a direct replacement of fluorescent lighting and issues surrounding light uniformity within a shelf, as well as proper replication and experimental design, have not been fully addressed. To overcome these limitations, use of LED lights positioned immediately above the lids of culture vessels was reported [16]. However, this system can only be used with specialized culture vessels that require a custom culture rack and they are not available commercially. An open source design suited to meet specific requirements of research labs with scope for further improvement and adaptation are not available.

Commercial culture vessels are generally manufactured by injection moulding. However, injection moulding requires large upfront investment which makes prototyping new vessels with this process extremely expensive [17]. Additive manufacturing (AM), or 3D-printing, is a technology in which models can be designed using a variety of software and manufactured using techniques such as fused deposition modeling (FDM), stereolithography (SLA), and selective laser sintering (SLS). Due to mainstream and hobbyist adoption, 3D printers have recently become small, affordable, and user friendly. While these techniques are generally not well suited to large-scale manufacturing, they allow rapid prototyping and small scale production of specialized/customized parts. This technology allows researchers who are familiar with the problems of their system to develop problem-specific solutions that may not be known to manufacturers or may not be feasible as a commercial product. Recently, this technology has also been employed to make customized labware [18–20], customized reaction-ware with reaction components printed for various chemistry applications [21, 22], as well as medical simulation and education [18, 23]. While this technology has great potential to improve plant tissue culture systems for species-specific solutions, it has not yet been applied in this field.

The objective of the current study was to evaluate the potential of 3D printing to develop a more efficient, open source, modular culture system with independently controlled integrated LED lighting for research and commercial micropropagation. This was accomplished by designing, 3D printing, and evaluating a culture system with tunable RGB LED lighting such that each vessel has its own light source that can be independently controlled to allow proper replication and randomization. Following initial tests, the vessel was manufactured using injection moulding to facilitate larger scale evaluation and use of the system. This paper describes this process and demonstrates the utility of 3D printing to improve culture systems by comparing the growth characteristics of *Nicotiana tabacum* and *Artemisia annua* under different spectra of light with proper replication. Here we present the first report of applying 3D printing technology for the design and development of a functional plant tissue culture vessel.

## Results and discussion

3D printing offers a cost-effective solution to manufacture and evaluate prototypes for in vitro cultures. This study provides a detailed demonstration of the procedure to produce/test FDM 3D printed vessels and devices for developing new systems to grow in vitro plant cultures and demonstrates their utility in conducting properly replicated experiments to study the effects of light on plant growth and development.

**Fig. 2:**
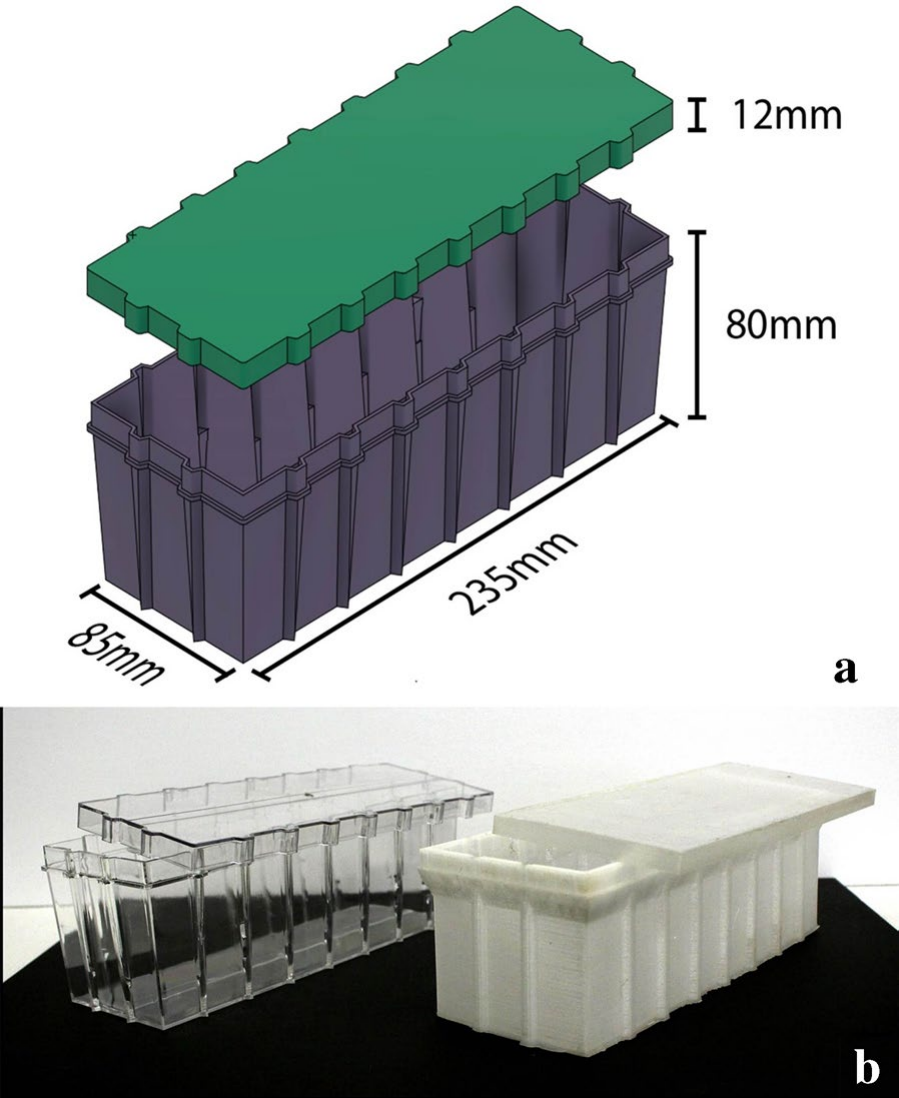
Dimensional drawing and design of culture vessels with lid and its 3D view before printing (**a**), and injection molded (*left*) and 3D printed culture vessels (**b**). Injection molded vessels was based on 3D printed design and produced following initial experiments

The culture vessel design depicted in Fig. 2 was developed to be compatible with both semi-solid culture and liquid based rocker systems [24–28], as well as being a suitable size to integrate commercially available RGB LED strips. One of the major limitations of FDM 3D printing with respect to plant tissue culture is that most materials currently used have relatively low melting points and are not suited to heat sterilization or autoclaving. However, while polycarbonate (PC) is not commonly used for 3D printing it is amenable to heat sterilization and has good optical clarity so was used in this study. Problems were encountered with this material related to warping, poor adhesion to the print bed, delamination between layers, and achieving water-tight prints. Warping and delamination were related in large part to poor adhesion to the build plate, and parts that did not stick well would inevitably fail. To improve build plate adhesion, several materials and adhesives were evaluated and the most effective combination was printing onto PolyEthylene terephthalate (PET) tape treated with a thin layer of disappearing purple glue stick (Elhmer’s Products, OH, USA). Another factor that was critical for successful printing with PC was accurate bed leveling and optimizing the height of the first layer. Warping was further reduced by printing with a fully enclosed 3D printer that helped create more uniform temperatures and even cooling of the molten plastic. Once these factors and the slicing parameters in the software were optimized (see Table 1), PC vessels and lids were successfully printed with minimal warping or delamination. However, when the vessels were tested for water tightness, many of them failed and water leaked through the bottom. This was addressed by adjusting the z-axis origin such that the first layer was closer to the build plate. By making this adjustment, the first layer slightly over-extruded to create a water-tight seal and 12 fully functional vessels were printed for pilot experiments as demonstrated in Additional file 2: Video S1.

**Table 1:** 3D printer parameter for vessels, lid and LED strips holder

3D parameters	Vessel	Lid	LED strips housing lid
Printer	AW3D AXIOM	AW3D AXIOM	AW3D XL
Filaments	PC	PC	PLA
Infill (%)	15	15	20
Layer height (mm)	0.3	0.3	0.3
Extruder temperature	310	310	200
Build plate temperature	140	140	60
Extruder speed while extruding (mm s^−1^)	60	60	60
Extruder speed while travelling (mm/s)	120	120	150
Model weight (g)	162	26	89
Model volume (cm^3^)	135	22	71
Model cost ($, approx.)	4.86	0.78	2.05
Print time (h, approx.)	7	1	3

Polycarbonate: 1.2 g cm^−3^, PLA: 1.25 g cm^−3^

**Fig. 3:**
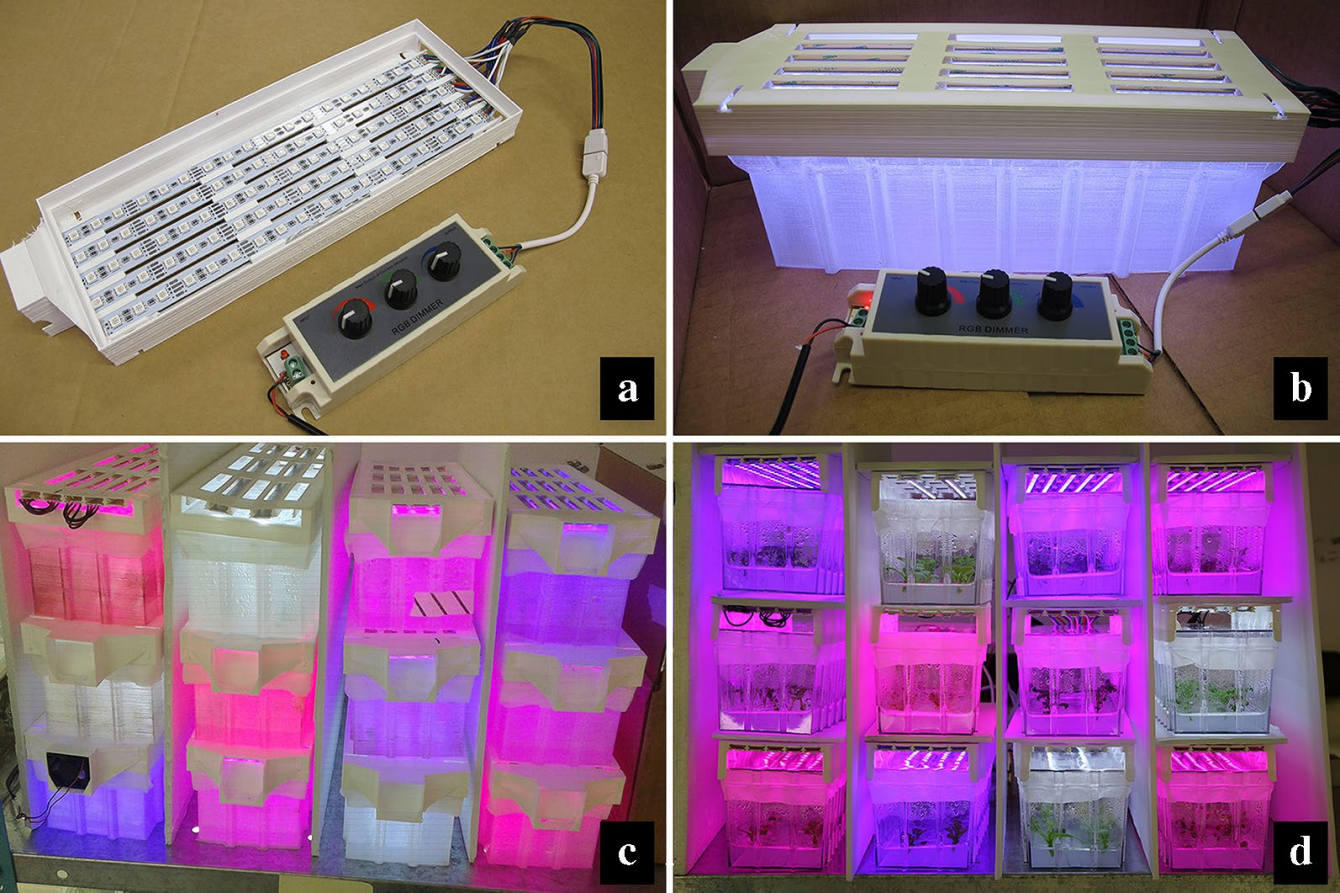
3D printed lid housing which is used to hold LED RBG strips and connector for power supply with provision for exhaust fan (**a**), whole assembly on 3D printed culture vessel (**b**). Several 3D printed units set at different light spectra stacked in a completely randomized design (**c**) and injection moulded culture vessels set at different light spectra stacked in completely randomized design (**d**)

For the secondary lids that held the LED strips (Fig. 3) heat sterilization was not required, so they were printed using polylactic acid (PLA). This posed no technical difficulties and 12 lids were manufactured to hold either tunable RGB or full spectrum white LED strips. Lids equipped with five RGB LED strips were capable of producing light fluence rates of approximately 225 μmol m^−2^ s^−1^. In initial tests this fluence rate caused the temperature to increase to 29 °C from an ambient temperature of 23 °C. Installing a fan in the optional fan slot as shown in Fig. 3a reduced the internal temperature to 27 °C. Light intensity for general plant tissue culture ranges from 25 to 50 μmol m^−2^ s^−1^ [29–33], but there is no universal standard [34–37]. At the fluence rate used in this study, 35 μmol m^−2^ s^−1^, there was no noticable increase in temperature and the optional fans were not used. This system facilitated experiments to evaluate the effects of light spectra on in vitro plant growth using a randomized complete block design with four treatments and three blocks on a single shelf, thereby providing sufficient replications for proper statistical analysis (Fig. 3). Using a traditional tissue culture system, this experiment would have required 12 culture shelves and would generally not be practical.

**Fig. 4:**
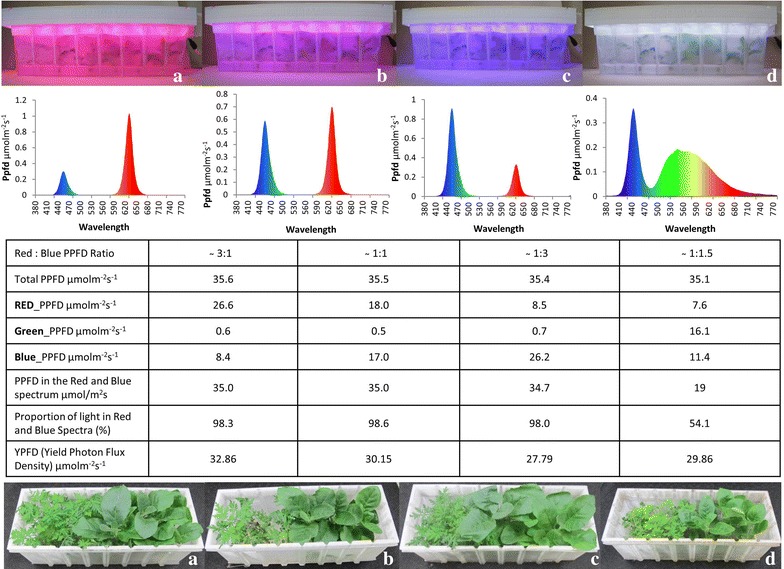
Tobacco and Artemisia plants cultured under in vitro condition with lid having various light spectra: **a** *red*:*blue* 3:1, **b** *red*:*blue* 1:1, **c** *red*:*blue* 1:3, **d** *white* with their respective graphs and fluence rate data and showing growth after 3 weeks period

**Fig. 5:**
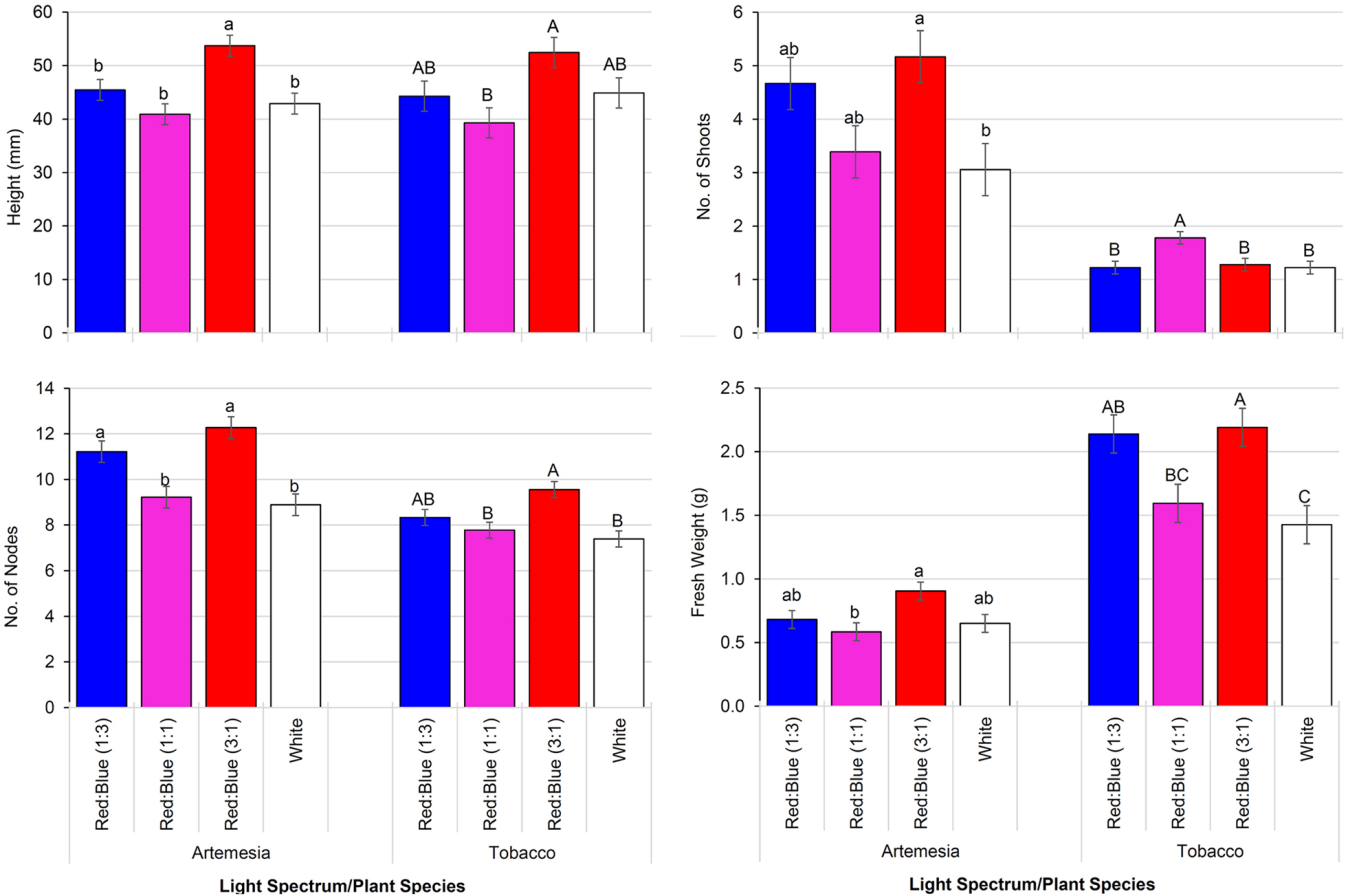
Differences in plant height, no. of shoots, no. of nodes and fresh weight measured after 3 weeks of growth of tobacco and artemisia growing under *white* and *red/blue* combination with the fluence rate 35 μmol m^−2^ s^−1^. Data presented as mean ± SE and *different letters in the figures* indicate significant differences at α = 0.05 using Tukey’s test (*lower* and *upper case letters* are used for artemisia and tobacco, respectively)

Different light spectra with similar overall fluence rates (~35 μmol m^−2^ s^−1^) significantly affected the growth of *N. tabacum* and *A. annua* plants (Figs. 4, 5). In general, plants cultured under red/blue light at a ratio of 3:1 performed the best (Figs. 4, 5). Compared to tobacco plants cultured under full spectrum white light, plants grown in red/blue (3:1) were of similar height (although many had reached the top of the containers such that height measurements may be skewed) and produced a similar number of shoots, but had a higher number of nodes and produced over 30% more fresh biomass. In the case of *A. annua*, somewhat different trends were observed between full spectrum white and red/blue 3:1, with plants grown under red/blue (3:1) being significantly taller, producing more shoots and nodes, but the overall fresh weight was not significantly different. Interestingly, the effects of red/blue 1:1 was similar to full spectrum white light with the exception that there were significantly more shoots produced in tobacco, while red/blue (1:3) produced results more similar to red/blue (3:1). These results highlight both the importance of light spectrum in plant growth and development and the fact that this response is species specific.

In general, this study agrees with the photosynthetic action spectrum in plants [38, 39] which indicates higher efficiency of red and blue light in driving photosynthesis. Goins et al. [40] observed photosynthetic rates and stomatal conductance in wheat leaves were increased under red-LED supplemented with blue light. It is generally acknowledged that this combination enhances plant growth and development by increasing net photosynthetic rate [9, 41]. The results observed in the current study are similar to previous work in a variety of species: Birch [42], *Cymbidium* [43], Lilium [44], southern pine species [45], Chrysanthemum [46], *Withania somnifera* [47], *Doritaenopsis* [48], *Phaelaenopsis* orchid [49] and lettuce [9] in which plant growth and development were affected by light quality in similar manners. Nhut et al. [35] have cultured strawberry plantlets under different blue to red LED ratios and compared its growth to that under plant growth fluorescent. The results suggest that a culture system using LED is advantageous for the micropropagation of strawberry plantlets and that it improved success in acclimatization, presumably due to increased photosynthetic capacity.

**Fig. 6:**
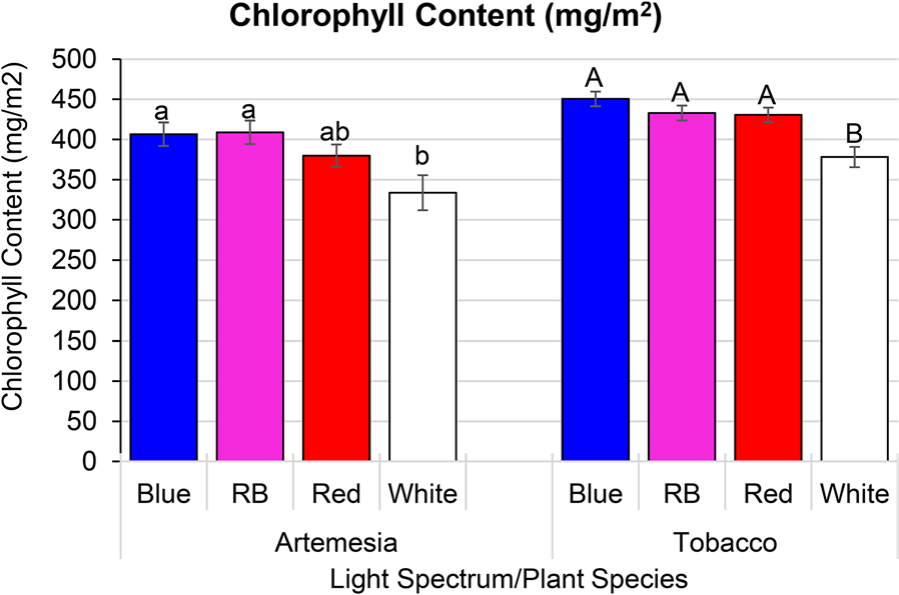
Average chlorophyll content of artemisia and tobacco plant 3 weeks of growth under different *red*/*blue* light combination and white with the fluence rate 35 μmol m^−2^ s^−1^. Data presented as mean ± SE and *different letters in the figures* indicate significant differences at α = 0.05 using Tukey’s test (*lower* and *upper case letters* are used for artemisia and tobacco, respectively)

While the effects of light spectra on plants growing photo-autotrophically can be relatively easily explained by increased photosynthetic efficiency, plants growing in vitro are heterotrophic and rely heavily on the sugars in the medium as a carbon source. In the current study, differences in chlorophyll content in *Artemisia* and tobacco plants were statistically significant among treatments, suggesting that the increased plant growth is due, at least in part, to photosynthetic capacity (Fig. 6). In both species, plants grown under white light contained significantly less chlorophyll than plants growing in red/blue (3:1) or red/blue (1:1). The effects of light quality on chlorophyll content agrees well with studies with lettuce, spinach, and birch [42, 50, 51]. However, Yorio et al. [52] reported that photosynthesis was not enhanced in leaves of lettuce under red-LED light supplemented with blue light. As such, it is unclear whether the increase in plant growth was a result of photosynthetic capacity, a physiological response leading to increased sugar uptake/use, or a combination of the two. Likewise, while the increased shoot production and number of nodes in some treatments may suggest that the light has signalling capacity, it is also possible that the plants were at a different physiological stage of growth at the time as a result of growth rates. It is also important to note that the Fv/Fm ratios were not significantly different among the treatments (Table 2) and indicated that none of the plants were under substantial stress that would interfere with proper growth.

**Table 2:** Average Fv/Fm ratio (max. quantum yield) ± SE after 3 weeks plant growth of Artemisia and tobacco with different light spectra

Light spectrum	Fv/Fm (max. quantum yield)	
	Tobacco	Artemisia
Red:blue (1:3)	0.875 ± 0.00354	0.875 ± 0.0034
Red:blue (1:1)	0.873 ± 0.0019	0.871 ± 0.00084
White (full spectrum)	0.870 ± 0.0000	0.875 ± 0.00354

While several questions remain unanswered in relation to the effects of light spectrum and fluence rate on in vitro plant growth, this system provides an ideal platform to address such questions with proper replication and statistical rigour. This culture system allows vessels to be stacked and the lights are at a close proximity to the plants, thereby using space and energy more efficiently and could increase overall productivity in a commercial setting. Based on the manufacturers’ specifications and measured light fluence rates, the LEDs lids would require about 32% less energy than the fluorescent tubes per μmol m^2^ s^−1^ delivered to the plants. However, it should be noted that the energy of LEDs varies among diodes, and that further energy savings may be possible with existing technologies. The development of this system was facilitated using 3D printing and vessels have now been injection molded for larger scale manufacturing. While this system currently only has the capacity to control three wavelengths of light, it is easily conceivable to develop a more advanced system using more LEDs to facilitate more precise spectral control. This demonstrates the utility of 3D printing to enable researchers familiar with existing limitations to improve upon existing systems, which will undoubtedly have a significant impact on plant tissue culture and other fields of research.

## Methods

### Light quality measurements

Light quality measurements (fluence rate and spectra) to test the uniformity of light quality over the area of the traditional culture shelf [53] were made at 84 positions evenly distributed in a grid over the horizontal plane of the shelf below the fluorescent lamps using light spectrometer (USB 2000+, Ocean Optics Inc.) (Fig. 1). This culture shelf had dimensions of 120 × 60 × 40 cm with two florescent bulbs (34 W per bulb) mounted on a ballast at the center. Light measurements were recorded at a distance of 31 cm every 10 cm across the length and width of the shelf in a grid formation. The spectral reads were analysed using Colour Calculator software (Osram Sylvania, Inc.). The fluence rate (Fig. 1) and CCT values (Additional file 1: Figure S1) are expressed as μmol m^−2^ s^−1^ and K units, respectively.

### 3D printed vessels with lid

Vessels with lid were produced using AW3D HD2X or AXIOM 3D printers (Airwolf_3D_, CA, USA) and PC filament (Fly Thinking Material Co Ltd., China). All units were designed using SketchUp or Fusion 360 (Autodesk) software and exported as STL (StereoLithography) files. The STL files (Additional files 3, 4, 5, 6) were processed using MatterControl 3D printing software and exported as gcode files. The dimension of the box was 235 L × 85 W × 80 mm H with a lid height of 12 mm (Fig. 2a). The box was designed with corrugations on side to give more strength and reduce warping (Fig. 2b). All printing parameters are shown in Table 1.

### 3D printed accessory lid

Accessory lids that hold the LED strips were produced using an AW3D XL 3D printer (Airwolf_3D_, CA, USA) with PLA filaments (Fly Thinking Material Co Ltd., China). The lids were designed (Additional files 5, 6) to hold five aluminium LED strips and a small fan in a similar way as mentioned above (Fig. 3). Small slits were developed to inset tabs in the four corners which allows space for air circulation between two units when it stacked. All printing parameters are shown in Table 1.

RGB strips holder assembly: five rigid RGB strips (LED light tech, China) were slid into small tracks built into the lid. Each strip had a total of 18 LED chips (5050 2.5 M, 0.2 W per LED). A three channel pulse-width modulation (PWM) controller (2010ourlonging, China) used to adjust RGB manually. The strips were connected in parallel to the PWM controller and 12 V DC power supply. All three units of the same treatments were connected to a single controller and power supply (Fig. 3). Additionally, each of these lids were designed such that they could also be used to illuminate three magenta boxes (a culture vessel widely used in vitro propagation), increasing the utility of the lid.

Injection moulded vessels and lids: after completion of the initial experiments, moulding tools were prepared with similar design and dimension for large scale production of vessels and lid (Kshama, Gujarat, India) and tested for culture in the same way as previously described (Fig. 2).

### In vitro plant growth using 3D printed vessels

In vitro-grown *N. tabacum* (tobacco) and *A. annua* plantlets were obtained from the germplasm collection at the Gosling Research Institute for Plant Preservation (GRIPP), University of Guelph, and multiplied on MS basal salt mixture with vitamins (PhytoTechnology, Shawnee Mission, KS, USA), 3% sucrose, and 2.2 g/L phytagel (PhytoTechnology, Shawnee Mission, KS, USA). The pH was adjusted to 5.75 prior to autoclaving at 121 °C and 118 kPa. These in vitro plantlets were clonal cultures obtained from single nodal explants and established under in vitro condition. Six explants of each plant from 4 weeks old shoots were transferred to 3D printed vessels containing the same medium. The maximum fluence rate using five LED strips was nearly 225 μmol m^−2^ s^−1^ with light spectrum red:blue:green (0.58:0.66:1.76). Cultures were kept at PPF of 35 μmol m^−2^ s^−1^ with a 16 h day^−1^ provided by RBG LED strips or full spectrum white (control) LED strips (LED light tech, China). Three sets of lids representing three replications were connected with the same controller and power supply and all boxes were randomly stacked (Fig. 4). LEDs lids remained on the top of the lid with some gap that allows the air circulation and LED lid have provision for a fan in the lid (Fig. 3a) which will helps in maintaining temperature in the case of high light intensity. Each vessel was separated with sheets of foam insulation between them to prevent light leaks. Light spectrum and fluence rate were adjusted for each lid using a portable spectrometer (model: lighting passport standard pro, make: Allied Scientific Pro, ON, Canada) as shown in Figs. 3 and 4. The light intensity measurements were recorded after the spectrometer gave a stable reading. The measurements were done over several averages of complete on–off cycles, over a period of time. Total four spectra selected for experiment viz., red:blue (3:1), red:blue (1:1), red:blue (1:3) and full spectrum white (Fig. 4). Observations were recorded shoot height, no. of shoot, no. of nodes and fresh weight after 25 days of culture.

### Chlorophyll content

The chlorophyll content of the in vitro leaves were estimated using a modulated ratio fluorescence chlorophyll fluorometer (CCM-300, Opti-Sciences, Hudson, NH, USA) based on the method developed by Gitelson et al. [54]. The results are expressed as chlorophyll content (mg m^−2^) and reported as mean ± SE.

### Kinetic imaging of chlorophyll fluorescence

Chlorophyll fluorescence kinetics assay was performed on dark adapted (>48 h) plantlets using a chlorophyll fluorescence imaging system (Z200 Open FluorCam, Qubit Systems Inc., Kingston, ON, Canada). The numeric data from the fluorescence measurements was used to compute the physiological parameters affecting the efficiency of PSII. The results are expressed as mean ± standard error for each of the parameters reported.

### Statistical analysis

The data from both the plant species were subjected to one-way analysis of variance (ANOVA) separately using JMP Pro 11.0.0 software (SAS Institute Inc, Cary, NC, USA). All statistical analyses were conducted using JMP version 10 (SAS Institute Inc. Cary, NC, USA). The mean values were compared using pairwise Tukey’s test at α = 0.05 significance level and the data is represented as mean ± SE. Treatments showing statistically significant difference are indicated by different letters in the graph (lower and upper case letters are used for Artemisia and tobacco, respectively).

## Additional files

**Additional file 1: Figure S1.** Variation in spectra of light across the shelf area represented by contour plot / heat map of Correlated Colour Temperature (CCT) values over a shelf.

**Additional file 2: Video S1.** Timelapse video for printing culture vessels using AXIOM 3D printers (Airwolf_3D_, CA, USA).

**Additional file 3.** STL (StereoLithography) file was designed for culture vessel (box) using SketchUp or Fusion 360 (Autodesk) software and the STL file was processed using MatterControl 3D printing software and exported as gcode files.

**Additional file 4.** STL (StereoLithography) file was designed for culture vessel's lid using SketchUp or Fusion 360 (Autodesk) software and the STL file was processed using MatterControl 3D printing software and exported as gcode files.

**Additional file 5.** STL (StereoLithography) file was designed for an accessory lid with fan slot using SketchUp or Fusion 360 (Autodesk) software and the STL file was processed using MatterControl 3D printing software and exported as gcode files.

**Additional file 6**. STL (StereoLithography) file was designed for an accessory lid without fan slot using SketchUp or Fusion 360 (Autodesk) software and the STL file was processed using MatterControl 3D printing software and exported as gcode files.
