# Supplementary figures and images for: Application of 3D printing to prototype and develop novel plant tissue culture systems
Source: Plant Methods. 2017 Jan 19;13:6. doi: 10.1186/s13007-017-0156-8 (PMC5244556; doi:10.1186/s13007-017-0156-8)

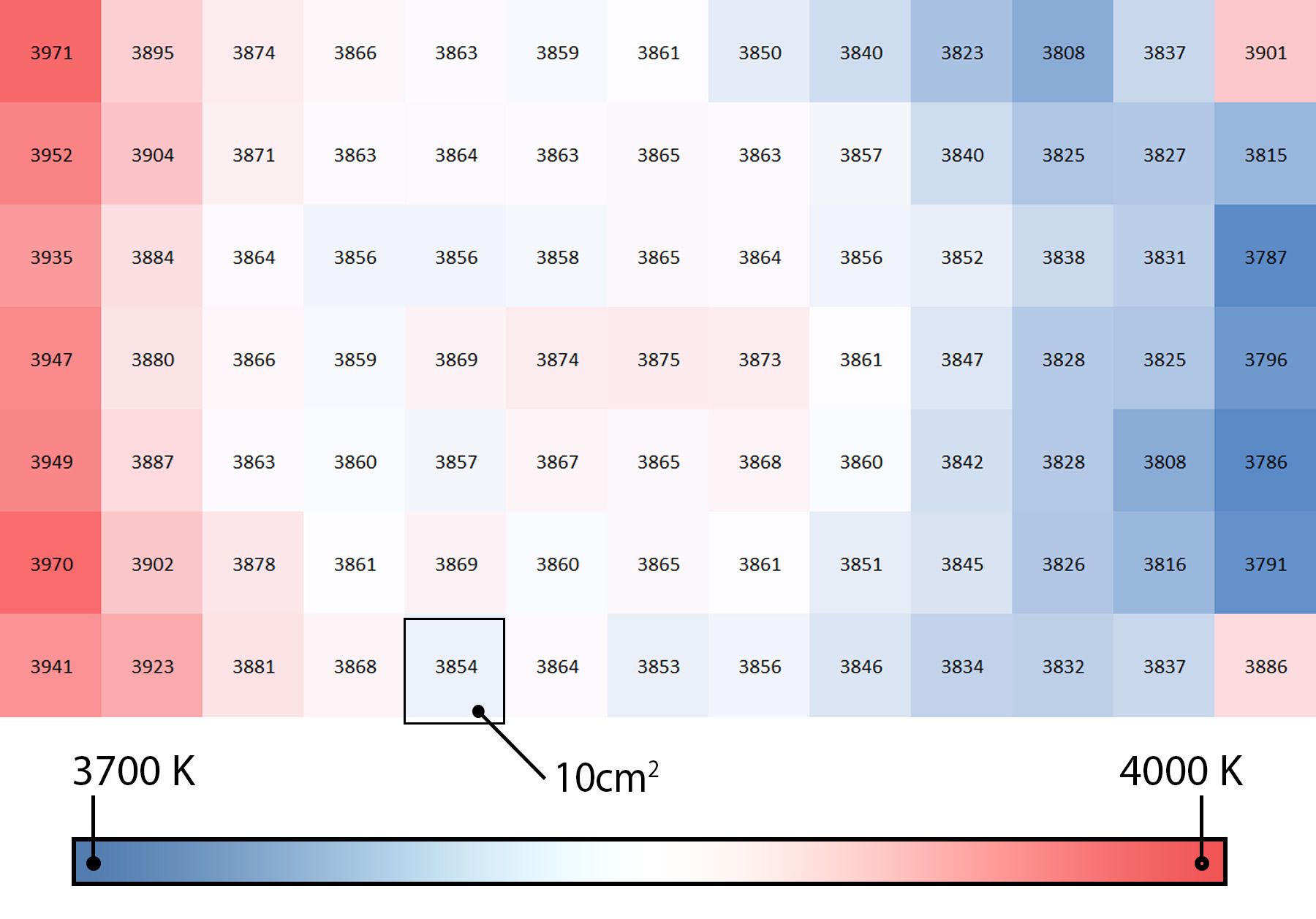

Supplement: Supplementary file 1 — Additional file 1: Figure S1. Variation in spectra of light across the shelf area represented by contour plot / heat map of Correlated Colour Temperature (CCT) values over a shelf. [file 13007_2017_156_MOESM1_ESM.jpg]
